# Biotemplated CdS Nano-Aggregate Networks for Highly Effective Visible-Light Photocatalytic Hydrogen Production

**DOI:** 10.3390/nano12081268

**Published:** 2022-04-08

**Authors:** Jiao He, Hongli Zhou, Guo Xiao, Yongjuan Chen, Zhiying Yan, Jiaqiang Wang

**Affiliations:** School of Chemical Sciences & Technology, National Center for International Research on Photoelectric and Energy Materials, Yunnan Province Engineering Research Center of Photocatalytic Treatment of Industrial Wastewater, School of Materials & Energy, Yunnan University, Kunming 650091, China; hejiao@ynu.edu.cn (J.H.); 13095319762@163.com (H.Z.); 15708537006@163.com (G.X.); chenyongjuan@ynu.edu.cn (Y.C.); zhyyan@ynu.edu.cn (Z.Y.)

**Keywords:** biotemplate, photocatalysis, hydrogen production, hydrilla, CdS

## Abstract

In the last few decades, many new synthesis techniques have been developed in order to obtain an effective visible-light responsive photocatalyst for hydrogen production by water splitting. Among these new approaches, the biotemplated synthesis method has aroused much attention because of its unique advantages in preparing materials with special morphology and structure. In this work, *Hydrilla verticillata* (L. f.) Royle was used as a biotemplate to synthesize a CdS photocatalyst. The as-synthesized sample had the microstructure of nano-scaled aggregate networks and its activity for photocatalytic hydrogen production was six times higher than that of CdS synthesized without a template in an Na_2_S-Na_2_SO_3_ sacrificial system. The use of Pt and PdS as cocatalysts further improved the hydrogen production rate to 14.86 mmol/g·h under visible-light (λ ≥ 420 nm) irradiation, so the hydrogen production can be directly observed by the naked eye. The results of characterization showed that the as-synthesized CdS photocatalyst has a high specific surface area and narrow band gap, which is favorable for light absorption and photocatalytic reaction. This work provides a new way to search for efficient visible-light catalysts and confirms the uniqueness of a biotemplated synthesis method in obtaining specially structured materials.

## 1. Introduction

The development and utilization of clean energy is an important way to solve the problems of energy shortage and ecological environment deterioration all over the world. Among the many types of new energy, hydrogen has attracted much attention because of its high calorific value and environmentally friendly use process. However, hydrogen used today is still mainly derived from processes that require the consumption of fossil minerals. Meanwhile, hydrogen production by photocatalytic splitting of water can directly convert solar energy into chemical energy. If large-scale and stable production can be achieved, it will have great development prospects [1,2]. In addition to the lack of research on reaction equipment and large-scale application, the low efficiency of photocatalysts is still an important obstacle in this field. In particular, the current most used photocatalysts are still based on semiconductors. Moreover, the utilization efficiency of the visible part (λ ≥ 420 nm) of the sunlight is very low because the band gap of some semiconductors is too wide. Therefore, the research of visible-light responsive photocatalysts has always been a hot topic.

On the other hand, the biotemplated synthesis method developed in the last decade or two shows its unique advantages in the synthesis of inorganic materials. It replicates the morphology and biological function of the template, or employs biological structures to drive the assembly of inorganic materials. Natural biomaterials often have unique, fine, and complex structures, which are difficult to obtain by traditional synthesis methods. Biotemplated synthesis provides a way to obtain materials with specific properties through an environmentally friendly process. The synthesis of semiconductor photocatalysts by biotemplating has also been widely used and obtained many good results [3,4,5,6]. In the previous works, we have some experience in the preparation of inorganic materials, especially metal oxide materials by biotemplating. TiO_2_ based on plant skin [7], diatom [8] and cyanobacteria [9] were successively prepared and applied to photocatalytic degradation of organic pollutants in water.

CdS has a narrow band gap, so it is easily excited by visible-light irradiation, but it also has the disadvantage of poor stability [10]. It is worth exploring whether biotemplated synthesis can improve its performance. However, the preparation of metal sulfide is a challenge when compared with metal oxide materials, mainly because it is difficult to remove the template material after preparation. In recent years, some biological templates, such as bacteria [11,12], enzymes [13], fungi [14], ferritin [15], bacterial S-layer proteins [16] and viruses [17,18], have been reported to be used to prepare CdS, but there are few studies on the catalytic and other application properties of these materials. It has been reported that *E. coli* biotemplated CdS hollow microrods exhibit excellent performance for photocatalytic hydrogen production, and the properties of the CdS nanomaterials could be tailored over a wide range by simply changing the synthetic conditions and the growth condition of *E. coli* [19]. A CdS nanoparticles/bacterial cellulose hybrid also demonstrated high-efficiency photocatalysis of methyl orange (MO) degradation and good recyclability [20]. In our previous work, *Hydrilla verticillata* (L. f.) Royle was used as biotemplate for synthesis of ternary sulfide ZnIn_2_S_4_ [21]. ZnIn_2_S_4_ with hexagonal-cubic phase junctions was obtained and it significantly enhanced the photocatalytic hydrogen evolution under visible-light in comparison to those of hexagonal or cubic single-phase structures. More recently, CdS nanowires with photocatalytic ability were obtained by using a tobacco mosaic virus as a biotemplate [22], the electrical properties of biomolecule DNA-templated nanowires-CdS/Au have been investigated [23], and Paulownia elongate tree leaves were used as a biotemplate to construct a sheet-like leaf structure black TiO_2_/CdS hetero-structure for photocatalytic hydrogen production [24]. Meanwhile, template methods using other microstructured substances are widely used to construct special CdS structures, such as CdS hollow microspheres [25,26,27]. This demonstrates the advantages of the templating method in the synthesis of CdS materials. Based on the works mentioned above, we believe that the use of a biotemplated method to synthesize more sulfide photocatalysts is worthy of further study. However, at present, many types of biotemplates have not been used for the synthesis of CdS photocatalysts, let alone for the study of visible-light photocatalytic hydrogen production.

*Hydrilla verticillata* (L. f.) Royle (referred to as “hydrilla”) is a common submerged plant found in many countries around the world. Its leaves are flexible and capable of efficient photosynthesis in an underwater environment, making it unique in light-harvesting [28]. Some studies have used the leaves of green plants to prepare a TiO_2_ photocatalyst with special morphology, which can produce hydrogen efficiently by water splitting under visible light [29,30]. Biomimetic synthesis was therefore successfully introduced into the field of photocatalysis. In this work, we use hydrilla as a biotemplate to prepare the CdS visible-light photocatalyst, the obtained CdS nano-aggregate networks were characterized carefully to reveal their physicochemical properties. Its visible-light photocatalytic activity for hydrogen production were evaluated. The effect of employing PdS and Pt as cocatalysts on the performance of CdS photocatalyst was also investigated.

## 2. Materials and Methods

### 2.1. Materials and Synthesis

Hydrilla (*Hydrilla verticillata* (L. f.) Royle) was purchased from Yuanbo market in Kunming, China. All the chemical reagents were of analytical grade and used as received. In a typical synthesis process, 100 g hydrilla was washed with distilled water and cut into 1 cm segments. Then, the hydrilla templates were immersed in Cd(NO_3_)_2_ (Sinopharm Chemical Reagent Co., Ltd., Shanghai, China) solution (500 mL, 0.1 M), exposed to ultrasonic for 1 h and aged for 24 h. After that, Na_2_S (Xilong Scientific Co., Ltd., Shantou, China) solution (100 mL, 0.5 M) was added followed by ultrasonic treatment for 6 h and additional aging for 24 h. The hydrilla templates were then separated from the liquid. The resultant suspension was filtered to obtain solid CdS sample. It was washed thoroughly, dried and labelled as CdS(HZ). Bare CdS synthesized with the same method in the absence of biotemplate was labelled as CdS(no template).

The CdS(HZ) catalyst was loaded with Pt and/or PdS co-catalysts before use through photo deposition and an in situ deposition process, respectively [31]. Generally, Pt was loaded through photoreduction of H_6_PtCl_6_ (Sino-Platinum Metals Co., Ltd., Kunming, China) under Xe lamp irradiation, while PdS was obtained in situ by reducing PdCl_2_ (Sino-Platinum Metals Co., Ltd., Kunming, China) with Na_2_S (Xilong Scientific Co., Ltd., Shantou, China). The sample loaded with 0.5% Pt in weight was labelled as Pt-CdS(HZ). The sample loaded with 0.5% Pt and 0.15% PdS in weight was labelled as Pt-PdS/CdS(HZ).

### 2.2. Characterizations

Wide angle powder X-ray diffraction (XRD) experiments were conducted on a Rigaku TTRIII diffractometer (Rigaku Co., Tokyo, Japan) with Cu Kα radiation. Diffraction patterns were collected at 2θ degrees, from 10° to 90°. Scanning electron microscopy (SEM) images of the samples were taken on an FEI Quanta 200FEG microscope (FEI, Hillsboro, OR, USA). The morphologies of the samples were examined with transmission electron microscopy (TEM) on a JEM-2100 microscope (Japan Electron Optics Laboratory CO., Ltd., Tokyo, Japan) with an accelerating voltage of 200 kV. Surface areas, pore volumes and pore size distributions were measured by the nitrogen adsorption/desorption measurements using a Micromeritic TriStar II 3020 surface area and porosity analyzer (Micromeritics, Norcross, GA, USA). The samples were degassed at 373 K for 3 h prior to analysis. X-ray photoelectron spectroscopy (XPS) was recorded using a Thermo Fisher Scientific K-Alpha^+^ XPS system (Thermo Fisher Scientific Inc., Waltham, MA, USA) with Al Kα radiation. UV-Vis diffuse reflectance spectra were measured on a Shimadzu UV-2600 photometer (Shimadzu Corp., Kyoto, Japan) from 200 nm to 800 nm. The photoelectrochemical measurement was performed using an CH660 electrochemical analyzer (Chenhua Instrument, Shanghai, China) in a standard three-electrode cell. The working electrodes were made from a mixture of the catalyst with N, N-Dimethyl formamide (Fengchuan Chemical Reagent Co., Ltd., Tianjin, China) as binder and then dropped onto the FTO surface (0.5 × 0.5 cm). Platinum wire was used as the counter electrode, and an SCE as the reference electrode. Na_2_SO_4_ (Fengchuan Chemical Reagent Co., Ltd., Tianjin, China) aqueous solution (0.2 M) was used as the electrolyte. A 300 W CEL-HXF xenon lamp (China Education Au-light Co. Ltd., Beijing, China) was used as the light source.

### 2.3. Photocatalytic Studies

The photocatalytic reactions were carried out in a home-made glass reaction cell with quartz cover connected to a closed gas circulation, and the gas circulation was swept by high purity N_2_ before irradiation. A 100 mg photocatalyst was dispersed in 100 mL of aqueous solution containing 0.5 M Na_2_S and 0.5 M Na_2_SO_3_ (Xilong Scientific Co., Ltd., Shantou, China) as the sacrificial reagents. Then the suspension was exposed to a 300 W Xe lamp (CEL-HXF300, China Education Au-light Co. Ltd., Beijing, China) equipped with an optical filter (λ > 420 nm) to cut off the light in the ultraviolet region. The reaction solution was cooled to room temperature by a flow of tap water. The amount of hydrogen evolved was determined at an interval of 1 h with online gas chromatography (GC-4C, Shimadzu Corp., Kyoto, Japan), which was equipped with an MS-5A column and TCD detector. N_2_ carrier was employed.

## 3. Results and Discussion

### 3.1. Characterizations

**Figure 1:**
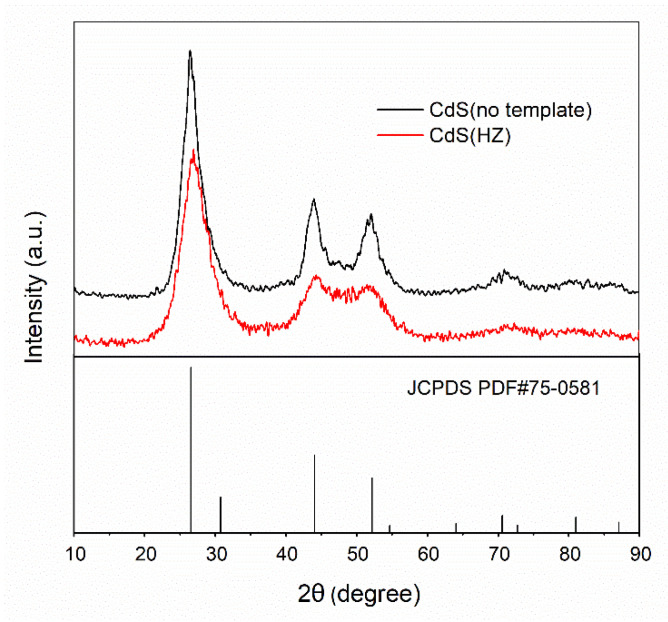
XRD patterns of CdS(HZ) and CdS prepared without template.

XRD pattern was obtained for CdS(HZ) and CdS(no template) to identify the crystallographic phase. As shown in Figure 1, the peaks at 2θ degree of 26.5°, 44.0°, 52.2° and 70.6° correspond to the diffractions of the (111), (220) (311) and (331) planes of hawleyite CdS (JCPDS 75-0581), respectively, confirming the cubic crystalline phase of the samples. No diffraction peaks of other impurities were detected. The peaks are all fairly broad, even overlapping with neighbors, such as the peak, due to (200) plane at 2θ degree of 30.8°, indicating the small sizes of the crystals that consisted of the CdS(HZ) and CdS(no template) [32,33,34]. The average crystallite sizes of different samples calculated using the Scherrer formula for the (111) facet diffraction peak is about 3 nm and 2.8 nm for CdS(HZ) and CdS(no template), respectively.

**Figure 2:**
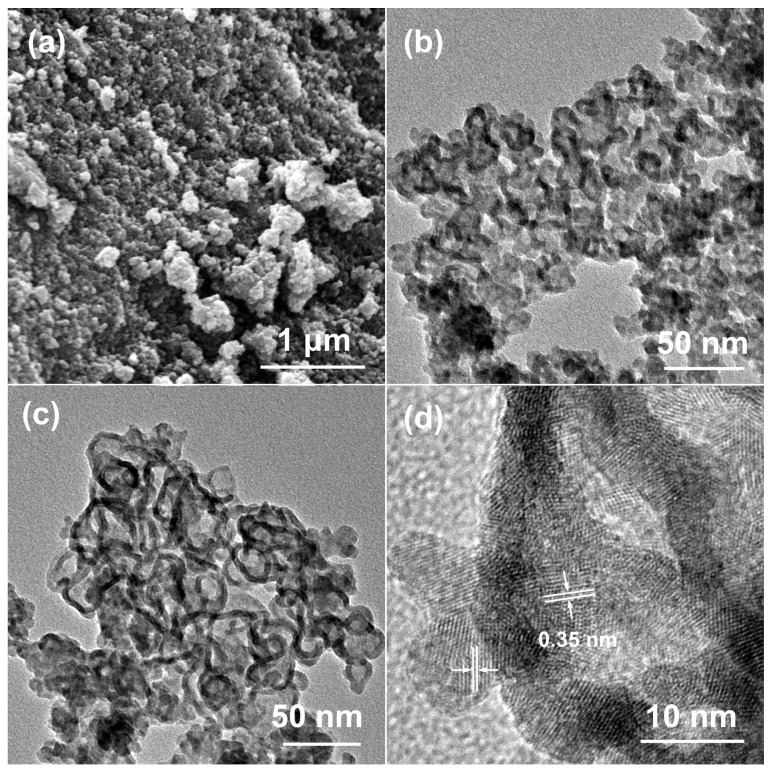
(**a**) SEM, (**b**,**c**) TEM and (**d**) HR-TEM images of CdS(HZ).

The morphology and structure of the samples were further investigated by SEM and TEM. The SEM image of the hydrilla biotemplate (Figure S1) shows a sheet of plant leaf tissue on which the cellular structure of the leaf can be seen. The SEM image (Figure 2a) shows that the CdS(HZ) is composed of aggregations of tiny particles and no specific morphology could be observed. However, TEM images reveal the nano-aggregate networks of the sample. As shown in Figure 2b,c, the CdS nanoparticles in a diameter of about 3 to 5 nm aggregate, weave and intertwine into the network, presenting many gaps, pores, and cavities. The size of the crystals is in good agreement with the result from XRD. Lattice fringes corresponding to (111) crystal planes of cubic CdS can be also distinctly observed (Figure 2d). This fiber-like structure, which could be observed in a CdS aerogel [35], can often maximum volume and surface area with a given material mass. In contrast, CdS materials synthesized without biotemplates (CdS(no template)) are blocks composed of small particles, and no other morphological characteristics of interest were observed (Figure S2). Obviously, the difference of morphology between the two samples is caused by the use of biotemplate. However, the morphology of CdS(HZ) is not similar to that of the template. Therefore, unlike ZnIn_2_S_4_ with a morphology similar to that of the biotemplate synthesized by using hydrilla in our previous work [21], the hydrilla template did not play the role of “hard template” when CdS was synthesized by using the method in this work. It is likely that the surface of the biological structure or some chemical substances in the bio-tissue regulate the growth of CdS crystals. This effect may be similar to the biomineralization process that is widespread in nature, the detailed mechanisms of which are unclear but worthy of study when constructing nanomaterials [36].

**Figure 3:**
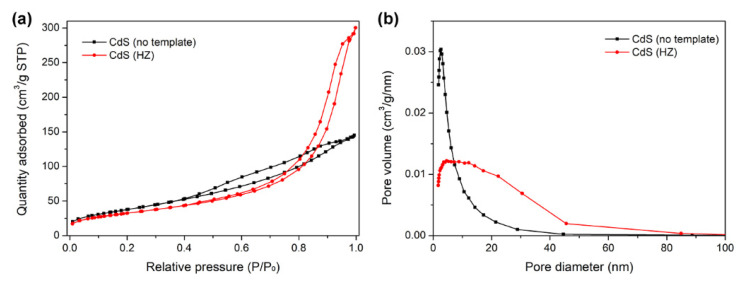
(**a**) N_2_ adsorption/desorption isotherm and (**b**) pore size distribution of CdS(HZ) and CdS prepared without template.

The N_2_ adsorption/desorption isotherm and pore size distribution of CdS(HZ) and CdS(no template) are illustrated in Figure 3. An IUPAC Type IV isotherm with a sharp capillary condensation step at high relative pressures and H1 type hysteresis loops are observed for CdS(HZ), which are characteristics of mesoporous materials. CdS(no template) also has a type IV isotherm, and its hysteresis loops is more likely to be H3 or H4 type. The BET (Brunauer–Emmett–Teller) surface area is determined to be 139.8 m^2^/g and 139.4 m^2^/g for CdS(HZ) and CdS(no template), respectively. This is a large surface area to CdS, even higher than many mesoporous CdS prepared by other methods, such as solid-state reaction [37], γ-ray irradiation [34], microwave-assisted synthesis [38], ultrasonic mediated precipitation [33], PVP templating [39], SBA-15 templating [40], aliphatic acids templating [41] and solution process at high temperature and pressure [42,43]. The large surface areas should be attributed to the gaps, cavities, and slits between the CdS nanocrystals because the samples are both composed by very few nanocrystals, as shown in Figure 2 and Figure S2. Considering that ultrasonic processing is used in the process of material synthesis, which can effectively prevent CdS crystals from growing up or agglomeration, so that the products are composed of very small particles. However, the total pore volume is 0.35 m^3^/g for CdS(HZ) while CdS(no template) only has a pore volume of 0.23 m^3^/g (Table S1). It can also be seen from the pore size distribution curve (Figure 3b) that the pore size of CdS(HZ) is widely distributed within the mesoporous range of 2–50 nm, while pore size distribution of CdS(no template) is more concentrated. Obviously, the special textural properties of CdS(HZ) are caused by the application of hydrilla as a biotemplate. These textural properties of CdS may be beneficial to photocatalytic reactions because more active sites are provided in comparison to bulk CdS.

In the processes of CdS synthesis, the use of ultrasonic treatment is mainly aimed at allowing cadmium and sulfur sources to penetrate into biological tissues and interact with various types of biological macromolecules. According to the results above, this process also effectively prevents the agglomeration of CdS crystals, so that the synthesized products have a small crystal size and a large specific surface area [44]. Meanwhile, it can be seen that the hydrilla biotemplate plays an important role in the process of CdS synthesis to form nano-aggregate networks. However, it is regrettable that the detailed mechanism of CdS crystal growth induced by hydrilla biotemplates is not clear at present. Since the size scale of CdS nano-aggregate networks is much smaller than that of hydrilla templates (Figure S1), it is reasonable to believe that in addition to the effects of biological tissue and cell, the presence of biological macromolecules in the template should also be an important reason to induce the formation of network structure. Although there have been many studies on the process of mineralization induced by biological materials [3,5], there are many kinds of biomolecules in the hydrilla biotemplates, which brings great difficulties to further confirmation. Even so, biotemplated synthesis still exhibits advantages and more possibilities than traditional chemical synthesis.

### 3.2. Photocatalytic Hydrogen Production

**Figure 4:**
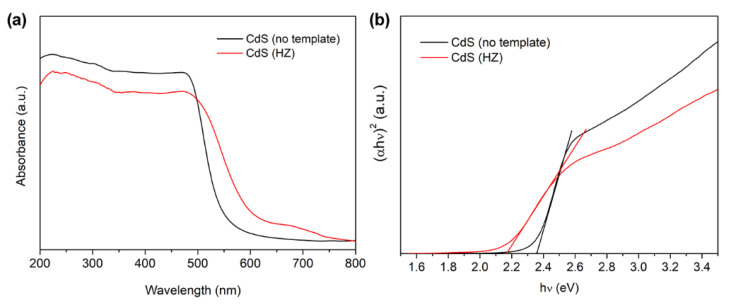
(**a**) UV-Vis DRS spectra and (**b**) energy band gap determination of CdS(HZ) and CdS synthesized without template.

The absorption and utilization of light by CdS synthesized by using a hydrilla biotemplate or not were investigated by UV-Vis diffuse reflectance spectra. As shown in Figure 4a, both CdS(HZ) and CdS(no template) have strong absorption in UV and visible-light regions, and the difference between them is mainly the location of absorption band edge. CdS(no template) shows the characteristics of ordinary CdS materials, with an absorption band edge of around 540 nm, corresponding to the 2.3 eV band gap [45,46,47,48]. Meanwhile, the absorption band edge of CdS(HZ) has a significant red shift to about 590 nm. In addition to nano-aggregate network morphology of CdS(HZ), residual biomolecules in the sample may also lead to red shift of the absorption edge and enhancement of light absorption in the visible region. The value of the optical band gap is calculated to be 2.37 eV and 2.17 eV, respectively, for CdS(no template) and CdS(HZ) by extrapolating the straight-line portion of (αhν)^2^ vs. hν graph, which has been shown in Figure 4b. This result indicates that CdS(HZ) may have a stronger ability to absorb and utilize visible light.

**Figure 5:**
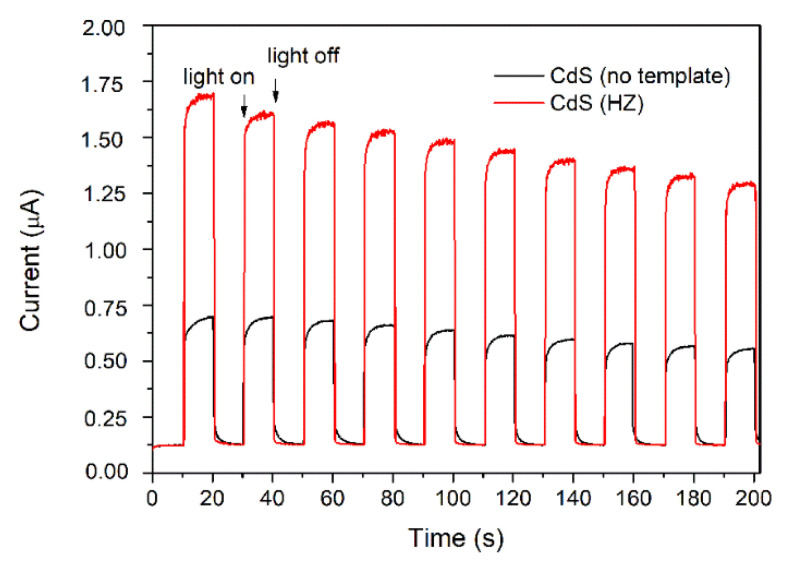
Photocurrent–time responses of CdS(HZ) and CdS prepared without template (350W Xe lamp as light source, in 0.2 M Na_2_SO_4_ solution).

In order to further investigate the effect of biotemplating on photoelectrochemical properties of CdS samples, the photocurrent responses of CdS(HZ) and CdS(no template) were tested. As can be seen from the photocurrent–time curve (Figure 5), the transient photocurrent density of CdS(HZ) is much higher than that of CdS(no template) under light illumination. According to the characterization results above, the as-synthesized samples are both made of closely contacted CdS nanocrystalline particles. However, CdS(HZ) has a special network structure and relatively higher visible-light absorption, so it might be able to become excited more efficiently by incident photons and the produced photo-generated carriers could be separated and transmitted more efficiently. In some studies, the photocurrent density of CdS is also different due to their differences in morphology and structure, such as CdS nanocrystals [49], CdS nanosphere and hollow CdS [27]. The use of biotemplates could also affect the photocurrent intensity of CdS [22].

**Figure 6:**
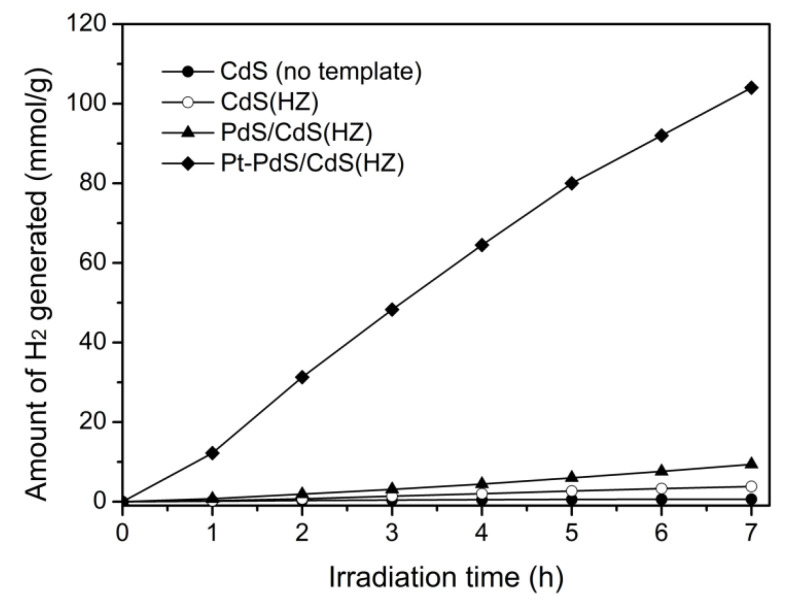
Photocatalytic H_2_ production over CdS, CdS(HZ), PdS/CdS(HZ) and Pt-PdS/CdS(HZ) under visible-light irradiation.

Photocatalytic hydrogen productions under visible light over the catalysts are shown in Figure 6. Bare CdS was synthesized with the same method to CdS(HZ), but no template was used. It has the lowest hydrogen production rate of 0.09 mmol/g∙h. When the hydrilla biotemplate was employed, CdS(HZ) exhibited a much higher activity of 0.55 mmol/g∙h, which is six times higher than CdS(no template). Therefore, the enhancement in photocatalytic activity could be attributed to the employment of a hydrilla biotemplate. The unique network structure and the enhanced visible-light absorption of CdS(HZ) derived from the biotemplate could be beneficial for photocatalytic reaction.

**Figure 7:**
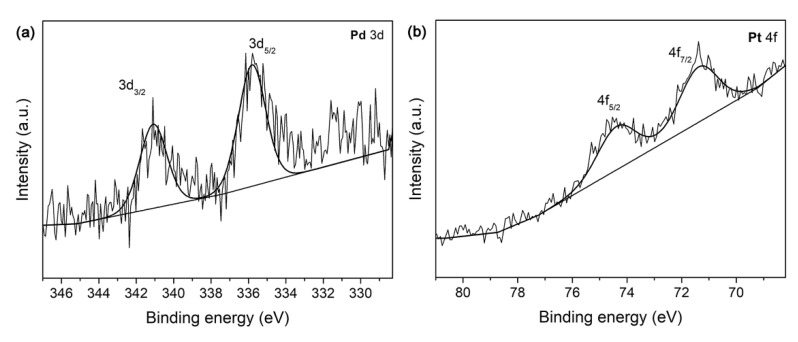
XPS spectra of Pt-PdS/CdS(HZ) in the (**a**) Pd 3d and (**b**) Pt 4f regions.

**Figure 8:**
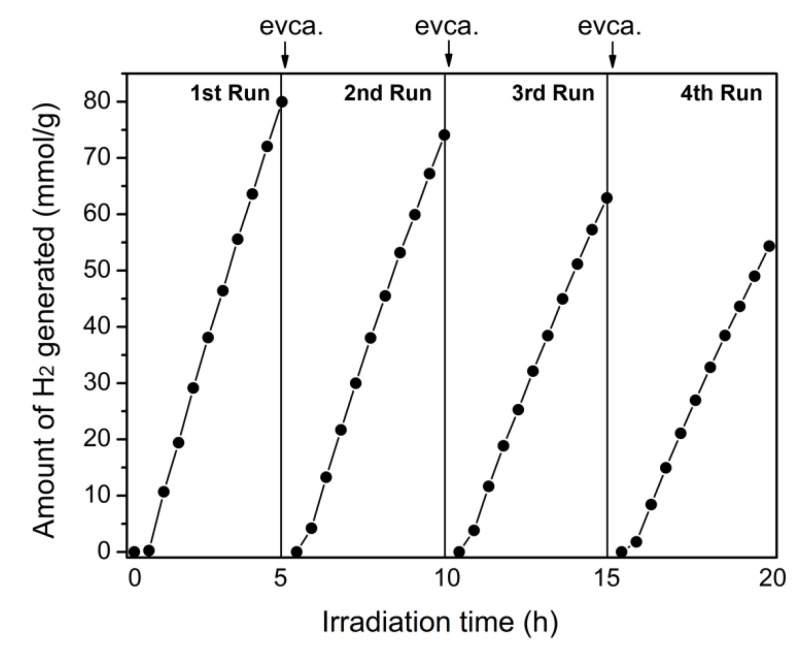
Stability of Pt-PdS/CdS(HZ) for photocatalytic H_2_ production over Pt-PdS/CdS(HZ) under visible-light irradiation.

However, whether hydrilla biotemplate was used or not during synthesis, the hydrogen production rates over pure CdS photocatalysts are not very high. Thus, PdS and Pt were loaded as oxidation and reduction cocatalysts, respectively, to improve the generation rates of hydrogen. High resolution X-ray photoelectron spectra (XPS, Figure S3) of Pd 3d and Pt 4f (Figure 7) verify the presence of Pd(II) and Pt(0) in Pt-PdS/CdS(HZ) [31,50,51], which confirms that the method for loading of PdS and Pt cocatalysts is reliable. When 0.15% PdS was loaded, the photocatalytic activity increased to 1.34 mmol/g∙h. Moreover, CdS(HZ) loaded with 0.15% PdS and 0.5% Pt achieved the highest hydrogen production activity of 14.86 mmol/g∙h. This is 27 times that of CdS(HZ). It is impressive that highly effective hydrogen production over Pt-PdS/CdS(HZ) under visible-light irradiation could be observed directly with the naked eye (Video S1). Moreover, the Pt-PdS/CdS(HZ) has a certain stability under illumination of visible-light in this system, as shown in Figure 8. At the fourth run of 5 h exposure to visible light, the amount of hydrogen generated decreased slightly, and HR-TEM images confirm that the nanocrystals of CdS(HZ) before and after photocatalytic reaction have no significant change (Figure S4).

**Scheme 1:**
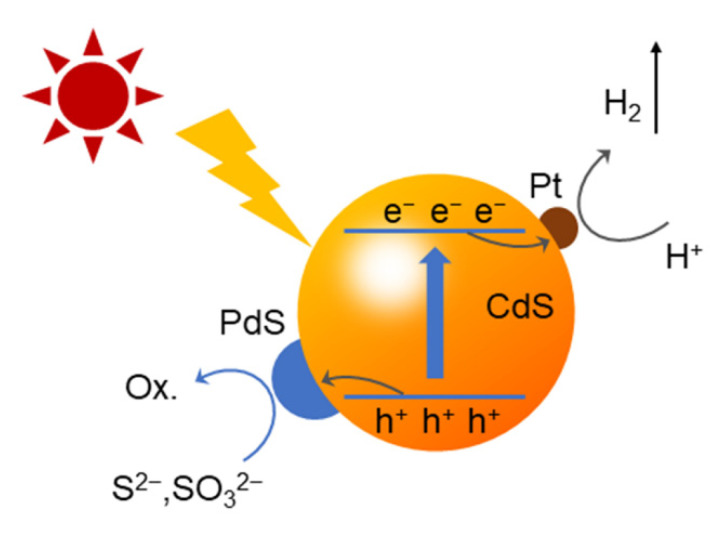
Proposed mechanism of photocatalytic H_2_ production over Pt-PdS/CdS(HZ).

The reasons for enhancement of photocatalytic hydrogen production over CdS photocatalyst has been proposed. Firstly, the use of a hydrilla biotemplate significantly narrows the band gap of CdS and enables more efficient absorption and utilization of visible light. Secondly, the CdS particles assembled to nano-aggregate networks. Such close contact between CdS nanocrystals may help to extend the life of photo-generated carriers [46,52]. These reasons make CdS(HZ) more active than CdS(no template) for photocatalytic hydrogen production. Then, the simultaneous loading of PdS oxidation cocatalyst and Pt reduction cocatalyst encouraged the separation and transfer of the photoexcited electrons and holes (Scheme 1). The noble metal deposits on the surface of the semiconductor photocatalyst and alters its electron distribution. Electrons are transferred from semiconductors with higher Fermi energy levels to Pt particles with lower Fermi energy levels, forming a Schottky barrier, which can effectively trap photogenerated electrons and prevent the recombination of photogenerated electrons and holes. On the other hand, PdS does not have the activity of photocatalytic splitting of water because the position of conduction band is higher than H^+^/H_2_ redox potential. However, PdS can be used as an oxidation cocatalyst to improve the photocatalytic activity of CdS [53]. Studies have shown that photogenerated holes generated by CdS can be transmitted to PdS. This could also inhibit photocorrosion of CdS, so that CdS can play a long-term and stable catalyst for water splitting [31]. Moreover, the large surface area of CdS(HZ) could also provide more reaction sites for photocatalytic reactions.

## 4. Conclusions

In summary, hydrilla was used as a biotemplate to synthesize a CdS photocatalyst for hydrogen production. The use of a biotemplate significantly enhanced the hydrogen production rate of CdS (six-fold increase). The unique nano-aggregate networks of CdS derived from the hydrilla biotemplate is supposed to be beneficial for photocatalytic reaction. The efficiency of visible-light photocatalytic hydrogen production has been greatly improved by using PdS and Pt as oxidation and reduction cocatalysts, respectively. A hydrogen production rate of 14.86 mmol/g∙h visible to the naked eye was achieved. This indicates that biotemplating synthesis has great potential in obtaining special morphologic nanomaterials and highly active photocatalysts.

## Data Availability

The data supporting the findings of this study are available within the article and its Supplementary Materials.

## Supplementary Materials

The following supporting information can be downloaded at: https://www.mdpi.com/article/10.3390/nano12081268/s1, Figure S1: SEM images of the biotemplate (*Hydrilla verticillata* (L. f.) Royle); Figure S2: SEM image of CdS synthesized without biotemplate (CdS(no template)); Figure S3: XPS Survey of Pt and PdS co-loaded biotemplated CdS (Pt-PdS/CdS(HZ)); Figure S4: HR-TEM images of CdS(HZ) (a) before and (b) after photocatalytic reaction. Table S1: Surface areas and textural properties of CdS(HZ) and CdS(no template); Video S1: Photocatalytic hydrogen evolution over Pt-PdS/CdS(HZ).
