# Supplementary material for: Biotemplated CdS Nano-Aggregate Networks for Highly Effective Visible-Light Photocatalytic Hydrogen Production
Source: Nanomaterials (Basel). 2022 Apr 8;12(8):1268. doi: 10.3390/nano12081268 (PMC9026159; doi:10.3390/nano12081268)
Supplement: Supplementary file 1 [file nanomaterials-12-01268-s001.zip › ESI.pdf]

Supplementary Materials

for

Biotemplated CdS Nano-aggregate Networks for Highly Effective Visible-light Photocatalytic Hydrogen Production

Jiao He, Hongli Zhou, Guo Xiao, Yongjuan Chen, Zhiying Yan and Jiaqiang Wang *

School of Chemical Sciences & Technology, National Center for International Research on Photoelectric and Energy Materials, Yunnan Province Engineering Research Center of Photocatalytic Treatment of Industrial Wastewater, School of Materials & Energy, Yunnan University, Kunming 650091, P.R. China; hejiao@ynu.edu.cn (J.H.); 13095319762@163.com (H.Z.); 15708537006@163.com (G.X.); chen-yongjuan@ynu.edu.cn (Y.C.); zhyyan@ynu.edu.cn (Z.Y.); jqwang@ynu.edu.cn (J.W.)

* Correspondence: jqwang@ynu.edu.cn; Tel.: +86 871 65031567

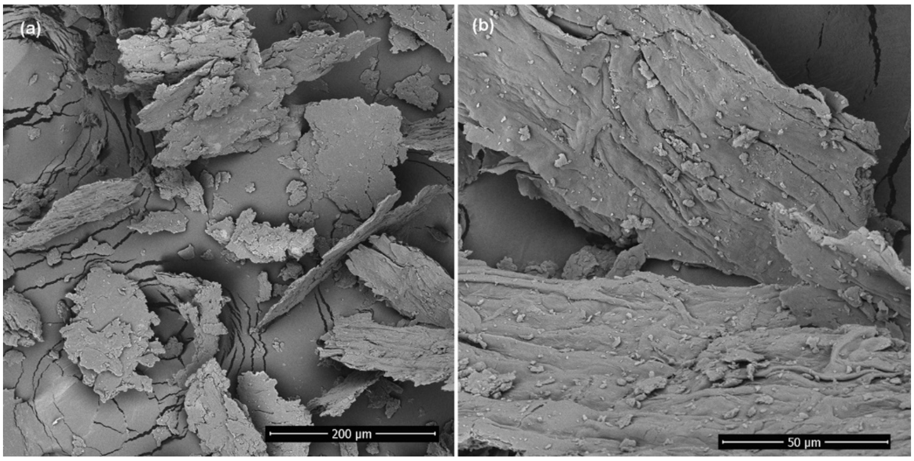

Figure S1. SEM images of the biotemplate (*Hydrilla Verticillata* (L. f.) Royle).

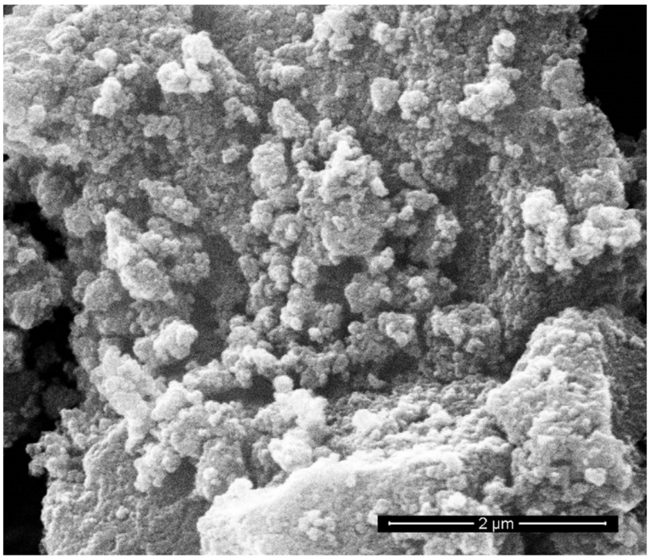

Figure S2. SEM image of CdS synthesized without biotemplate (CdS(no template)).

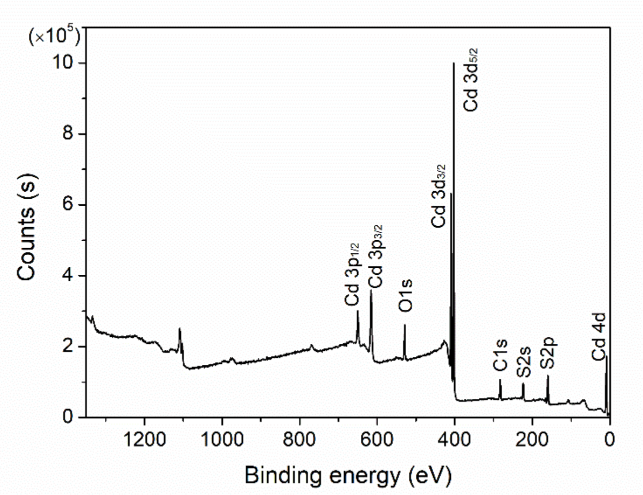

Figure S3. XPS Survey of Pt and PdS co-loaded biotemplated CdS (Pt-PdS/CdS(HZ)).

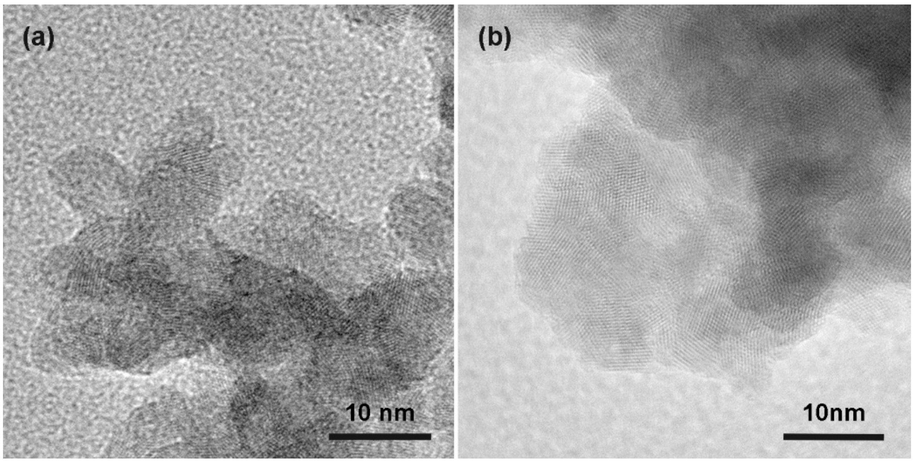

Figure S4. HR-TEM images of CdS(HZ) (a) before and (b) after photocatalytic reaction.

Table S1. Surface areas and textural properties of CdS(HZ) and CdS(no template).

**Sample**	S_{BET} (m^2/g)	Pore Volume (cm^3/g)	Average pore size (nm)
CdS(HZ)	139.8	0.35	8.3
CdS(no template)	139.4	0.23	5.0
